# Aristol

**Published:** 1891-01

**Authors:** 


### Aristol.

Aristol is the name of a new antiseptic introduced to take the place of iodoform, iodole, and zozo-iodole. Since iodoform has been shown by W. D. Miller, of Berlin, to be useless in the treatment of root canals of teeth, dental practitioners will naturally seek something to take its place.

Aristol was introduced into this country by W. H. Scheiffelin & Co., of New York, and is prepared by the Farbenfabriken, Elberfeld, Germany.

Aristol is in the form of a powder, which when rubbed in the hand feels like a resinous powder; being rather sticky it adheres to the flesh easily. Its color is an approach to the salmon color, and is comparatively inodorous. The freedom from disgusting odor like iodoform will make it a welcome visitor to the dental office which should not smell like an apothecaries' shop.

Aristol is a combination of iodine and thymol and is said to be very valuable. It retails at about two dollars per ounce, but weighing lightly, a small quantity will last a long time. Sixty grains will easily dissolve in one-half ounce of ether.

Perhaps the best way of using aristol is, make a solution of it in ether or chloroform, both of which readily dissolves it, and as an antiseptic, dressing it can be easily introduced, in that state, into root canals previous to filling.

Aristol is insoluble in water and will not easily dissolve in alcohol.

Oils containing fats will dissolve it. Heat will decompose aristol; so it is best to use it after the tooth has regained normal temperature after using the hot-air syringe.

This antiseptic may be especially recommended to the dental profession in the treatment of the results of actino-mycosis. It will not irritate, and is not poisonous.

The drug should be kept in dark-colored bottles, and only small quantities bought at a time, so as to insure freshness.

W.

---

Progressive Exercise in Practical Chemistry, by Henry Leffman, M.D., Ph.D., Professor of Chemistry in the Woman's Medical College of Pennsylvania, in the Pennsylvania College of Dental Surgery, and in the Wagner Free Institute of Science; Pathological Chemist of the Jefferson Medical College Hospital.

And William Beam, M.A., Demonstrator of Chemistry in the Pennsylvania College of Dental Surgery, Associate of the Society of Public Analysts of Great Britain. Illustrated.

This little work, recently published by P. Blakiston, Son & Co., is a very valuable aid to the beginner in the study of chemical science and art. It is especially valuable in laboratory work, at least so far as it pertains to inorganic chemistry. Experimental workers find in this work much help, not only in the text, but in the illustrations as well. Much attention is given to details in regard to quantity of materials to be used, and arrangement of apparatus.

Some of the experiments and forms of apparatus are new and have been devised especially with a view to economy; a matter of importance to all.

Formulæ and discussion of purely theoretical questions have been omitted. This is a valuable little manual for all, and especially for beginners. The matter is very much condensed, but is full of instruction.

The work may be obtained of any bookseller.

